# Estimating actual SARS-CoV-2 infections from secondary data

**DOI:** 10.1038/s41598-024-57238-0

**Published:** 2024-03-20

**Authors:** Wolfgang Rauch, Hannes Schenk, Nikolaus Rauch, Matthias Harders, Herbert Oberacher, Heribert Insam, Rudolf Markt, Norbert Kreuzinger

**Affiliations:** 1 Unit of Environmental Engineering, Department of Infrastructure, University of Innsbruck, Technikerstrasse 13, 6020 Innsbruck, Austria; 2 Interactive Graphics and Simulation Group, University of Innsbruck, Innsbruck, Austria; 3 Institute of Legal Medicine and Core Facility Metabolomics, Medical University of Innsbruck, Innsbruck, Austria; 4 Department of Microbiology, University of Innsbruck, Technikerstrasse 25, 6020 Innsbruck, Austria; 5 Department of Health Sciences and Social Work, Carinthia University of Applied Sciences, Villach, Austria; 6 Institute of Water Quality and Resource Management, Technical University Vienna, Vienna, Austria

**Keywords:** Epidemiology, Engineering, Scientific data

## Abstract

Eminent in pandemic management is accurate information on infection dynamics to plan for timely installation of control measures and vaccination campaigns. Despite huge efforts in diagnostic testing of individuals, the underestimation of the actual number of SARS-CoV-2 infections remains significant due to the large number of undocumented cases. In this paper we demonstrate and compare three methods to estimate the dynamics of true infections based on secondary data i.e., (a) test positivity, (b) infection fatality and (c) wastewater monitoring. The concept is tested with Austrian data on a national basis for the period of April 2020 to December 2022. Further, we use the results of prevalence studies from the same period to generate (upper and lower bounds of) credible intervals for true infections for four data points. Model parameters are subsequently estimated by applying Approximate Bayesian Computation—rejection sampling and Genetic Algorithms. The method is then validated for the case study Vienna. We find that all three methods yield fairly similar results for estimating the true number of infections, which supports the idea that all three datasets contain similar baseline information. None of them is considered superior, as their advantages and shortcomings depend on the specific case study at hand.

## Introduction

Key aspect in pandemic management is accurate information on infection dynamics to plan for timely installation of control measures and vaccination campaigns. Covid-19 surveillance relies to a huge extent on diagnostic testing of individuals (based on swab testing), thus reporting the key parameter confirmed cases on a given day. However, this number does not reflect the actual number of new infections on that date due to delays and uncertainties in the reporting system^1^. Most important is the underestimation of the true number of infections as (mostly asymptomatic) patients do not seek healthcare and thus are not accounted for by diagnostic testing^2^. In the following we denote the underestimation in the surveillance as underreporting and the missing cases as undocumented infections. Quantification of the undocumented cases and thus of total infection numbers is an important issue both for monitoring the effectiveness of institutional responses but also to understand the propagation of the epidemic in the population^3^. Underreporting not only results in biased estimates but also in misleading public perception of the severity of the pandemic.

Since underreporting can accurately only be determined by costly large-scale random screening studies, alternative methods to estimate true infection dynamics have been developed from the start of the pandemic and are included in national Covid-19 models^4^. Different strategies are pursued, e.g. by accompanying prevalence and seroprevalence surveys (e.g., Oran and Topol^5^), dynamic modelling of the infection dynamics (e.g., Rippinger et al.^6^) or estimates from secondary data such as test positivity rate and infection fatality rate^7–9^ etc. Likewise, capture-recapture methods based on documented infections and death counts have been successfully applied for estimation of underreporting^10^. Recently prevalence of total infections has also been computed from social media data by means of Google Trends^11^. Detailed literature reviews on the estimation of underreporting are given by Millimet and Parmeter^12^ and Mehraeen et al.^13^.

Wastewater-based epidemiology (WBE) as an alternative Covid-19 surveillance scheme collects the virus signal for a drainage system. The key idea is that each infected patient in the sewered area sheds a certain amount of virus load into the wastewater (mostly connected with stool but also due to sputum and other excrements—^14^). Since the monitored total viral load stems from the shed amount of viral RNA from all patients in the sewered area, the signal is a proxy for the total amount of infection cases. Consequently, if we can estimate the amount of RNA shed per individual infection case, we can derive information on the true number of infection cases in the watershed. This ability to estimate infection dynamics without underreporting is a key virtue of the surveillance method. Since the background of wastewater-based epidemiology (see e.g., Medema et al.^15^) as well as its application for prevalence estimation (e.g., Li et al.^16^; Gerrity et al.^17^) is described in the literature, we refrain from repeating this information and use the timeline of virus concentrations measured in the inflow to wastewater treatment plants as a starting point.

The aim of our paper is to derive a robust relation to estimate the true number of infections from the timeline of secondary data. As such data are easily accessible (most are publicly available) the relation allows for a simple and low-cost alternative to estimate underreporting. Capture-recapture methods are following the same principle^18^. The common parameter-less formulation of the approach^10^ allows for robust estimates in the absence of prevalence information but lacks in flexibility to adapt the model to changing conditions in the course of the pandemic. It is due to this shortcoming that the capture-recapture method proved to be unsuitable for the present investigation.

Accordingly, we investigate and compare three parameterized models, based on (a) test positivity, (b) case fatality and (c) the signal from wastewater-based epidemiology for this task. As case study we will use the situation in Austria from April 2020 to December 2022, thus covering nearly the whole entity of the pandemic occurrence. For calibration, the results of several prevalence and seroprevalence studies in the same period are used to generate (upper and lower bounds of) credible intervals for true infections. Model parameters are subsequently estimated by applying both Approximate Bayesian Computation and Genetic Algorithms. For validation, the models are used to estimate the prevalence for the city of Vienna, Austria.

## Materials and methods

We start with a brief definition of the key pandemic parameters in the context of this paper, followed by an overview of the available data, i.e. epidemic surveillance data, sero-prevalence study results and wastewater monitoring. Next, we present the three methods to estimate prevalence based on test positivity rate, infection fatality rate and wastewater monitoring, and last, we describe the Approximate Bayesian Computation scheme for parameter estimation as well as the application of Genetic Algorithms. No human participants are involved in the study but data has been provided by external laboratories or organisations. Neither protected data is used, and the investigation is carried out fully in accordance to guidelines and regulations.

### Incidence, prevalence and seroprevalence

Typically, pandemic management relies on diagnostic testing of individuals, reporting the number of positive tests on a given day *t* as documented daily new infection cases (*N*_*INF*_). In fact, there is a time lag between infection and testing that includes both the incubation period and the latency between symptom onset and testing^19^. However, as it has no influence on the derived methodology, we choose to disregard this time lag in the following—thus taking *N*_*INF*_ as reported. Note that this time lag can be easily introduced to the method (e.g., by adapting the input timeseries *N*_*INF*_) but adds additional parameter for the time shift.

The timeline of documented new infections (*N*_*INF*_) is denoted as incidence information and is a key information in pandemic management. However, here we are interested in the timeline of active infection cases (*I*—containing of both documented (*I*_*d*_) and undocumented (*I*_*u*_) ones) in the population. This is—different from above—a measure of prevalence, with Prevalence (*P*) defined as (point) ratio of infections in the population (*P* = *I*/*N*).

For addressing the ratio of persons who are immune against the disease (e.g., as already been infected) we use the term seroprevalence (*SP*) and define: *SP* equals the sum of persons with antibodies for the disease divided by the population. A common approach to determine *SP* is to sum up the daily new infection cases ($SP = \sum N_{INF} /N)$ which is also denoted as cumulative incidence. Note that this simple equation is correct only at the early stages of the pandemic: as antibodies are both waning with time (see e.g., Shioda et al.^20^) and increased due to vaccination (see e.g., Forgacs et al.^21^), antibodies no longer stringent indicate past infections.

### SARS-CoV-2 related data for Austria

Data from the surveillance program on individual cases (number of new infections *N*_*INF*_, number of Tests: *N*_*TEST*_) as well as associated public health data (recovered patients: *N*_*REC*_ and fatalities: *N*_*FAT*_) have been collected daily since the start of the pandemic by the Austrian Agency of Health and Food Safety (AGES) and is publicly available^22^. Documented active cases (*I*_*d*_) can be estimated therefrom by using cumulative numbers from the start of the pandemic, subtracting recovered patients and fatalities from documented cases^23^. However, the documentation of *N*_*REC*_ is considered to be unreliable and often just based on the estimate of a mean duration of infection^24^. Consequently, we estimate active cases by summation of positive tests over the mean infection time *t*_*inf*_ = 14 days (coinciding with the requested quarantine period in Austria), i.e. by applying cumulative incidence over 14 days.

1 $$ I_{d} \left( t \right) = \mathop \sum \limits_{{t^{*} = t - (t_{inf} - 1)}}^{t} N_{INF} \left( {t^{*} } \right) $$

Note that this (common) approach to determine active infections is to be regarded as a data filter and thus introduces a time shift, i.e., the signal of *N*_*INF*_ precedes the resulting infection *I* by the period *t*_*lead*_ $\approx \frac{{t_{inf} }}{2}$.

**Figure 1:**
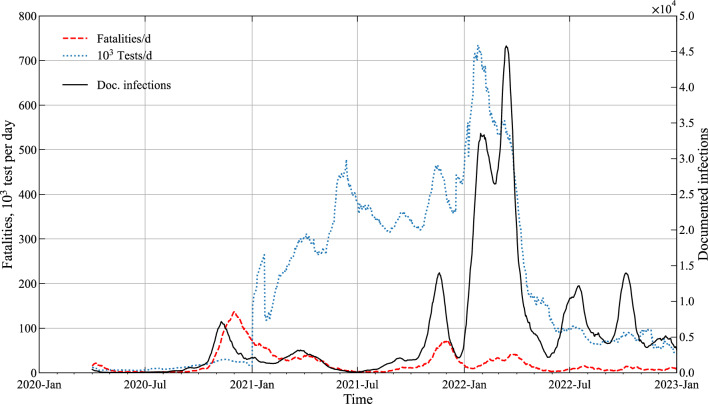
Timeseries of SARS-CoV-2 surveillance data—national situation in Austria. Fatalities (*N*_*FAT*_), number of Tests (*N*_*TEST*_) and number of documented new infections (*N*_*INF*_) are given as daily values, averaged over 7/21 days.

The timeline of the data in Fig. 1 specifies the Austrian situation on a national basis from the start of the monitoring in April 2020 to December 2022. Note that we apply a moving average smoothing filter to the data with a sampling width of 7 days for *N*_*INF*_, *N*_*FAT*_ and* I*_*d*_^25^. We use the same smoothing filter also for *N*_*TEST*_ but need to set the sampling width here to 21 days due to the high random fluctuations in the number of daily tests. It also has to be noted that the counting procedure of tests has been changed around January 1st, 2021 which introduces disturbances in the daily test data *N*_*TEST*_ around that period. Likewise, information on the occurrence of the dominant variants: Alpha, Delta and Omicron (see supplementary Fig. S1 online) is publicly available in a dashboard^22^ with Alpha starting in February 2021, Delta in June 2021 and Omicron in mid-December 2021.

Figure 1 visualizes the following aspects of the Austrian situation: (a) the number of daily tests was significantly increased starting with January 2021 to Spring 2022 and (b) the fatality rate during the Omicron wave has clearly dropped as compared to the earlier situation. Likewise, the occurrences of dominant variants since the beginning of 2021 are clearly visible as pandemic waves in the incidence data, i.e., in the number of daily new infections (*N*_*INF*_).

### Wastewater based SARS-CoV-2 monitoring

In Austria SARS-CoV-2 wastewater monitoring (i.e., RT-qPCR-based assessment of genome quantity) started early in the pandemic with the first reliable data available in April 2020. The number of monitored plants has been steadily extended, eventually covering > 70% of the population in 2021. Since January 2022 the National SARS-CoV-2 Wastewater Monitoring Program of the Austrian Federal Ministry of Social Affairs, Health, Care and Consumer Protection is in place. A detailed description on the monitoring data as well as the methodology is given e.g., in Daleiden et al.^26^; Amman et al.^27^; Markt et al.^28^; Schenk et al.^29^ and will henceforward not be repeated herein. For each treatment plant the resulting data is pretreated and normalized with the population marker NH_4_-N^25^.

**Figure 2:**
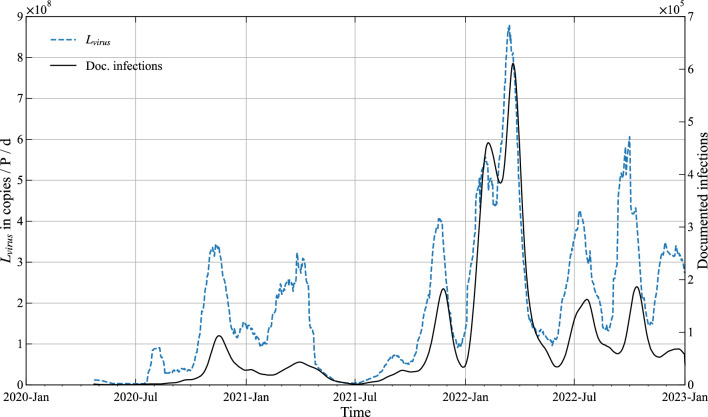
Timeline of wastewater samples expressed as virus load *L*_*virus*_ in 10^6^ gene copies per Person per day for Austria. For comparison active documented infections (*I*_*d*_) are plotted on the secondary axis.

As prevalence survey data (for model parameter estimation) is only available on a national basis, the wastewater signal is likewise to be compiled into a national one by computing a weighted average—based on plant design capacity. Note that the resulting national wastewater signal, displayed herein as virus load *L*_*virus*_ (for definition see below), is derived from results of several laboratories. The signal consequently contains uncertainties not only due to averaging on a national basis but also from differences in laboratory procedures and methods used. As the resulting timeline exhibits large random fluctuations, data smoothing is necessary. For consistency we apply also here a moving average smoothing filter with sampling width of 21 days (see Fig. 2). The comparison with the timeline of active documented infections (secondary axis in Fig. 2) reveals the correlation of the two signals, which is also documented in the literature^30^.

### Relevant prevalence data for Austria

For the peak of the second wave of the Covid-19 pandemic a prevalence study has been conducted for SARS-CoV-2 occurrences in Austria based on individual PCR diagnosis^31^. Prevalence was estimated for the period 12th–14th November 2020 as 3.1% (95% CI 2.6–3.5%). The underestimation in prevalence of the total infections as compared to documented cases—denoted in the following as prevalence ratio (*P*_*R*_ = *I*/*I*_*d*_)—is computed as 3.6, given appr. 78,500 documented cases and the Austrian population *N* = 9.02 × 10^6^.

Bicher et al.^32^ estimate total seroprevalence (*SP*_*tot*_) i.e., the sum of all infected persons until 1st February 2021 as 14.7% (95% CI 9.1–36.8%) based on an agent-based model that is used as forecast for the Austrian pandemic management. Assuming that documented seroprevalence equals the sum of recorded new infections at this moment ($SP_{d} = \sum N_{INF} /N = 4.53 \%$) results in a ratio of 3.2 for total versus documented seroprevalence.

A seroprevalence study among those persons in Austria that have prior been neither infected nor vaccinated has been conducted in the period 30^th^ November 2021 to 13th January 2022^33^. The study is assumed to be representative for the situation in December 2021, i.e., just before the Omicron variant started. Seroprevalence in that group has been determined as 21.7% (95% CI 17.6–25.4%). Assuming that this relation (*r*_*underereporting*_) of underreporting in seroprevalence is generally applicable gives the following relation for total seroprevalence: $SP_{tot} = \left( {1 - SP_{d} } \right)*r_{underreporting} + SP_{d}$. For *SP*_*d*_ = 14.1% at 31.12.2021 the ratio of total versus documented seroprevalence is computed as 3.05.

For the occurrence of the Omicron variant, there are no specific prevalence/seroprevalence studies available for Austria. However, a nationwide seroprevalence study in Germany in the period from November 2021 to February 2022^34^ evaluates the prevalence ratio as 1.5 to 2^35^. We assume that this ratio also holds for Austria and is likewise representative as a mean value for the later stage of the pandemic, i.e. the year 2022.

**Figure 3:**
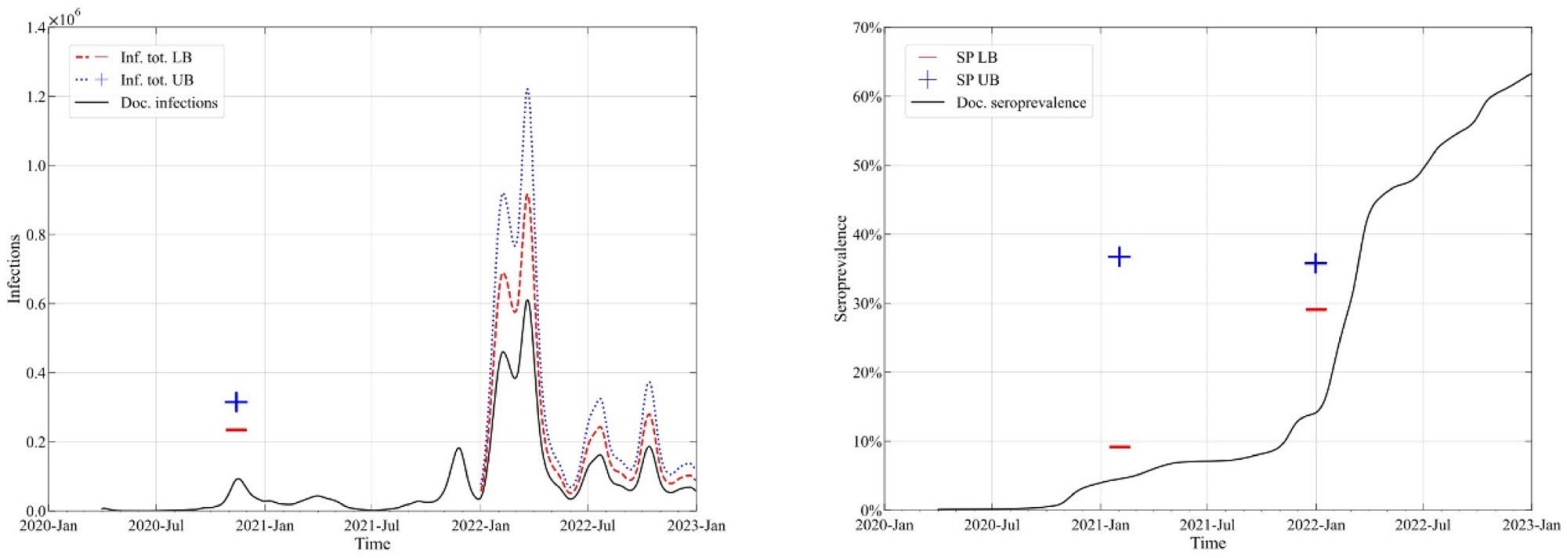
Upper and lower bounds of credible intervals for Left: total infection number and Right: total Seroprevalence for 2 data points each. The timelines of documented infections/seroprevalence are plotted for comparison.

Figure 3 depicts the upper (UB) and lower bounds (LB) of four credible intervals from the Austrian survey data. Left, the intervals are plotted for the total numbers of active cases and right, the intervals are given for total seroprevalence. The comparison of the credible interval with the timeline of documented cases/documented seroprevalence indicates the underreporting. Upper and lower bounds for the prevalence screening study in November 2020, the seroprevalence model result for 1st February 2021 and the seroprevalence study for 31st December 2021 are computed as 95% CI values (2.5% and 97.5%). We estimate the upper and lower bounds of infections for the Omicron (BA2) wave from the results of the seroprevalence study in Germany and use the information given by RKI, 2022 for the mean prevalence ratio in the period January to December 2022 as interval $\left[ {1.5, 2.0} \right]$.

### Prevalence estimation based on test positivity rate and reported cases

It is a well-known fact that the number of diagnostic tests is instrumental for the correct assessment of the pandemic development: the smaller the number of diagnostic tests, the larger the error. This is most easily seen in the relation of positive tests to the total number of diagnostic tests: if the relation is high (e.g., close to 1) a major part of the infection is likely missed—and underreporting is high. Accordingly, Chiu and Ndeffo-Mbah^36^ argue that the test positivity rate is correlated to the prevalence of undiagnosed infected persons by a time-dependent bias factor *b*

2 $$ P_{ + ,\tau } \left( t \right) = b\left( t \right) \times \frac{{I_{u} \left( t \right)}}{N} $$

where the test positivity rate is expressed here as *P*_+_ = *N*_*INF*_*/N*_*TEST*_, i.e. the number of new infections divided by the total number of tests for a given point in time *t* and *N* is the total population. Chiu and Ndeffo-Mbah^36^ further assume that the bias factor *b* is inversely related to the testing rate (*N*_*TEST*_/*N*) and define a convex (negative power) function:

3 $$ b\left( t \right) = \left[ {\frac{{N_{TEST} }}{N}} \right]^{ - n} $$

with $0 \le n \le 1$ (typically *n* $\approx$ 0.5). The above can be interpreted as follows: First, the higher the testing rate (the closer to 1) the smaller is the bias b and vice versa. Second, for the lower limit for *n* = 0 no bias occurs, thus resembling a random sampling situation, whereas for *n* = 1 the bias is sharply increased reflecting a situation where everyone infected is tested. Rearranging the above equations and introducing the expected time shift *t*_*lead*_ for the delayed occurrence of *I*_*u*_ as compared to the positive tests, we get for the timeline of undocumented infections:

4 $$ I_{u} \left( {t + t_{lead} } \right) = N_{INF} \left( t \right)*N_{TEST}^{n - 1} \left( t \right)*N^{1 - n} $$

The time shift* t*_*lead*_ is determined by cross correlation analysis (using *I*_*d*_ as proxy for *I*_*u*_) as 6 days. In the following we address this estimation of total infections (*I* = *I*_*u*_ + *I*_*d*_) as POS model as it uses both the timeline of positive tests and the total number of tests.

### Prevalence estimation by back-casting from reported fatalities

Given the number of fatalities *N*_*FAT*_ for a given day as well as the infection fatality rate (*IF*_*R*_), the occurrence of the initial infections can be estimated backwards in time. The total number of new infection cases at a given day (*C*_*INF*_) can be estimated straightforward by assuming that the time from infection to death (*t*_*death*_) is constant e.g., 14 days.

5 $$ C_{INF} \left( {t - t_{death} } \right) = \frac{{N_{FAT} \left( t \right)}}{{IF_{R} }} $$

However, it is obvious that *t*_*death*_ is not constant but varies according to personal constitution and infection severity. Flaxman et al.^37^ suggest that *t*_*death*_ follows a Gamma distribution (probability density function = *f*_*(x;a,b)*_) and compute the new infection numbers at a given day t’ from the fatalities at day t as

6 $$ C_{INF} \left( {t^{\prime } } \right) = \frac{{N_{FAT} \left( t \right)*f_{{\left( {t - t^{\prime } ;\alpha ,\beta } \right)}} }}{{IF_{R} }}. $$

Phipps et al.^38^ use this approach for back-casting and compute the total number of active infections (*I*) for each day by summing up these new infections (i.e., applying *Eq. *1). Details on the statistics of the approach and its implementation are given in the original paper and is not repeated herein. Following Phipps et al.^38^ we estimate the values of the Gamma distribution as α = 4.938 and β = 2.835 resulting in a mean *t*_*death*_ of 14 d (SD = 6.3 d). The model is denoted as FAT model as it relies on the timeline of fatalities.

Note that the basic information N_FAT_ is potentially underreporting the true number of deaths related to the infection^12,39^. While this introduces an additional source of uncertainty in the estimation of true infections the effect is compensated by calibration of the parameter IF_R_ in Eq. 6.

### Estimating prevalence from wastewater-based epidemiology

Wastewater based epidemiology (WBE) in the context of public health aims to derive information on the occurrence of pathogens in the watershed of a sewer system by sampling—usually at the influent of a treatment plant^40^. Adapting the basic formulation for the case of SARS-CoV-2, the (measured) virus load at the monitoring point is related to the population drained with the sewer system (for details on data preprocessing see e.g., Rauch et al.^25^):

7 $$ L_{virus} = \frac{{c_{virus} *Q}}{N} $$

where *L*_*virus*_ = virus load in gene copies/P/d; *Q* = flow volume in L/d; *c*_*virus*_ = virus concentration in the sample in gene copies/L and *N* = number of persons in the watershed. Assuming that each infected person is shedding a certain load of genetic material per time (*L*_*shed*_ in gene copies/P/d) into the sewer system as well as introducing a general loss term *f*_*loss*_ we get the relation:

8 $$ I\left( {t + t_{lead} } \right) = \frac{{L_{virus} \left( t \right) \times N}}{{L_{shed} \times f_{loss} }} = \frac{{L_{virus} \left( t \right) \times N}}{{L_{corr} }} $$

with *I* = infected persons in the watershed, *t*_*lead*_ = time lead and *f*_*loss*_ = dimensionless loss factor. *L*_*corr*_ is the corrected virus shedding load in gene copies/P/d. This approach is denoted in the following as WBE model as it uses the signal from wastewater-based epidemiology as input.

The lead time of the signal in the wastewater as compared to the occurrence of infection (documented infection *I*_*d*_) is determined by cross correlation analysis as *t*_*lead*_ = 7 d. This coincides with the results of e.g., Aberi et al.^30^ and Olesen et al.^41^.

The parameter *f*_*loss*_ stands for all losses and distortions of the virus signal in the transport phase, during sampling and analysis. This parameter is case specific and encompasses temporal and spatial variable phenomena such as virus transport in the sewer system, dispersion, sedimentation, resuspension, but also (temperature-dependent) degradation and loss via combined sewer overflow. Since also the viral load shed by an individual infected person (*L*_*shed*_) varies substantially both on an individual basis (depending on the constitution of the patient and the degree severeness of the illness) and along the timeline of the infection^14^ we use in the following *L*_*corr*_ indicating the corrected shedding load in gene copies/P/d. It is to be assumed that *L*_*shed*_ is not constant but varies with virus variants^42^ which consequently also applies to *L*_*corr*_.

### Key features of the prevalence models

The three models for estimating prevalence vary according to the number of data sets and parameters needed to compute true infection dynamics. The test positivity model (POS) uses two input data sets (daily new infections *N*_*INF*_ and number of tests *N*_*TEST*_) but only one parameter (*n*) that is assumed as time invariant. The infection fatality rate model (FAT) is based only on the timeline of daily fatalities *N*_*Fat*_ but the parameter *IF*_*R*_ varies with time. Similarly, the wastewater-based epidemiology model (WBE) uses only the timeline of the measured virus load, but the corrected shedding parameter *L*_*corr*_ needs to be adapted along the timeline. In the following a procedure is discussed to compute the parameter values based on the prevalence survey data presented in Fig. 3.

### Estimating model parameters with Approximate Bayesian Computation

Computational methods typically apply the following basic procedure for model parameter estimation: sample from a search space of parameter values θ and determine those that give the best fit with the measured data *D*. However, in the given problem setting we do not have unique measured data but instead credible intervals for data values (see Fig. 3). Bayesian inference allows to include uncertainty and probability to the parameter estimation. In this framework (see Gelman et al.^43^) the posterior distribution of the parameters given the data $p\left( {\Theta |D} \right)$ is computed by the likelihood $p\left( {D|\Theta } \right)$ and the prior distribution of the parameters $p\left( \Theta \right)$ using Bayes’ theorem:$p\left( {\Theta |D} \right) \propto p\left( {D|\Theta } \right)p\left( \Theta \right)$.

The shortcoming of this approach is the estimation of the likelihood that is at least computationally expensive (see e.g., Gelfand and Smith^44^). Approximate Bayesian Computation (ABC) methods circumvent that issue and approximate the likelihood function by a comparison between the observed and the simulated data^45^. The most basic form of ABC schemes is the rejection sampler^46^ which involves the following steps in a Monte Carlo simulation context:

- Sample a parameter θ from a given a priori distribution of values $p\left( \Theta \right)$
- Compute a dataset D^*^ by applying θ to the model
- Reject θ if D^*^ is too distant from measured D—otherwise accept

After a sufficiently high number of samples drawn, a subsample of accepted parameter values θ is derived which is approximately distributed according to the posterior distribution $p\left( {\Theta |D} \right)$. The key advantage of ABC is the avoidance of the complex evaluation of the likelihood function and wide range of applicability which made the method quite popular in recent years^47^.

In the context of our aim, we apply basic ABC sample rejection to determine the parameters of the three models presented above that are based on secondary data i.e., (a) test positivity (b) infection fatality and (c) wastewater monitoring. For each sampled parameter θ (or set of parameters) we compute the timelines of estimated total infections $\hat{I}$ and total seroprevalence $\widehat{{SP_{tot} }}$. The parameter is accepted if $\hat{I}$ and $\widehat{{SP_{tot} }}$ are within the credible intervals for the 4 data points.

Note that more refined and advanced ABC schemes are available (e.g., Marin et al.^47^, Sunnåker et al.^48^) but not necessary for the problem at hand. It is actually the ease of including rejection criteria that makes this basic scheme the preferred option. For increasing computational efficiency, the ABC algorithm is coded directly in ANSI C. Sample number was chosen as 10^6^ which yielded stable results.

### Estimating model parameters with genetic algorithms

For testing the results of the ABC scheme, we additionally apply standard parameter estimation by error minimization with a Genetic Algorithm (GA)^49^. For the error function we cannot use the credible intervals directly but need to convert the information into a continuous function for each survey. We start by assuming that the true infections (*I*) within the 95% confidence interval [*I*_*LB*_,*I*_*UB*_] are normal distributed: ${\text{\rm I}}\sim {\text{\rm N}}\left( {\mu ,\sigma^{2} } \right)$ with $\mu = \frac{{I_{LB} + I_{UB} }}{2}$ and $\sigma \sim \frac{{\mu - I_{LB} }}{2}$. For each parameter estimation we compute the estimated total infections $\hat{I}$ at the survey point and with the transformation $z = \frac{{\hat{I} - \mu }}{\sigma }$ the density $\varphi \left( z \right) = \frac{1}{{\sqrt {2\pi } }}e^{{ - \frac{1}{2}z^{2} }}$. By scaling with standard normal distribution $\varphi \left( 0 \right) = \frac{1}{{\sqrt {2\pi } }}$ the error function for one survey is defined as $e = \frac{{\left| {\varphi \left( z \right) - \varphi \left( 0 \right)} \right|}}{\varphi \left( 0 \right)} \in \left[ {0,1} \right]$. We formulate similar for the total prevalence *SP*_*tot*_ and compute the total error as sum of *e* for all 4 credible intervals.

The GA is binary coded and implemented in ANSI C according to^50,51^. The population size is set as 1000 with 100 generations.

## Results

### Parameter estimation

Three quite distinctive approaches have been presented above to estimate prevalence based on different sets of secondary data. For all three models we assume only one parameter each as variable (*n, IF*_*R*_* and L*_*corr*_), while all others are seen as constant values (e.g., lead time or gamma distribution values). But while *n* (POS model) is assumed to be time invariant, this does not apply for *IF*_*R*_ (FAT model) *and L*_*corr*_ (WBE model). The value and occurrence of both is influenced by the occurrence of SARS-CoV-2 variants.

In their paper, Phipps et al.^38^ assume *IF*_*R*_ to be constant with 0.76% (95% CI 0.37–1.15%). While this was correct for the early stages of the pandemic, *IF*_*R*_ has declined with time. Reed. et al.^52^ determine for Austria IF_R_ as 0.404% (95% CI 0.214–0.75%) at 15th October 2020 and 0.386% (95% CI 0.205–0.745%) at 1st January 2021. But *IF*_*R*_ dropped substantially with the onset of the Omicron variant—in Austria in the beginning of January 2022^53^. Nyberg et al.^54^ estimate that *IF*_*R*_ during Omicron is reduced to 0.31% of Delta values. For parameter estimation we thus assume two parameters for the FAT model with *IF*_*R*_*_1* reflecting the situation until 17th December 2021 and *IF*_*R*_*_2* the Omicron variant since 1st January 2022. In order to avoid unrealistic step changes in the parameter values we apply a linear transition for IF_R_ in between these dates (14 days).

No quantitative information is available for the variation of *L*_*corr*_ but Puhach et al.^42^ describe three different phases for viral shedding, i.e., (a) Ancestral (before variants) with lowest viral load (b) Delta with highest load and (c) Omicron with viral load in between. Our data suggests that shedding in Austria is approximately similar in the Ancestral phase and the phase after the first Omicron (BA1) wave, i.e., from May 2022 onwards. As indicated by Puhach et al.^42^, the shedding load during the Alpha and Delta variants (most of 2021) was certainly higher. However, and contradicting Puhach et al.^42^, according to the Austrian data the shedding load in the first Omicron wave (BA1) is significantly smaller. Accordingly, we use three parameters to describe shedding dynamics in the WBE model, *L*_*corr*_*_1* for the early stage of the pandemic until 1st February 2021 (Ancestral) as well as for the period from 1st May 2022 onwards, *L*_*corr*_*_2* for the period of Alpha and Delta variants (15th February 2021 until 15th December 2021) and *L*_*corr*_*_3* for the first Omicron wave (1st January 2022 until 16th April 2022). Again, we apply a linear transition to the parameter values over the 14 days in between the indicated dates in order to avoid unrealistic step changes.

Table 1—upper part—gives the information on the parameters used, most important the time variance (application) for *IF*_*R*_*_1 and IF*_*R*_*_2* as well as for *L*_*corr*_*_1, L*_*corr*_*_2 and L*_*corr*_*_3.* Likewise, Table 1 states the upper-lower bounds for the prior parameter value distribution in ABC and GA with the value range estimated from the literature (see above discussion). The prior distribution of the parameters $p\left( \Theta \right)$ is then estimated as uniformly distributed in the interval: lower–upper boundary.

**Table 1:** Parameters of the three models POS, FAT and WBE with parameter *L*_*corr*_ in log_10_ units.

	POS model	FAT model		WBE model (log_10_ values)		
	n	IFR_1	IFR_2	L_corr__1	L_corr__2	L_corr__3
Lower boundary	0.30	0.40	0.03	9.80	10.00	9.80
Upper boundary	0.60	0.80	0.10	10.20	10.40	10.20
Application	Total series	Until 17.12.2021	From 1.1.2022	Until 1.2.2021 and from 1.5.2022	From 15.2.2021 until 17.12.2021	From 1.1.2022 until 16.4.2022
Result 5%	0.391	0.540	0.065	10.080	10.150	9.900
Result 50%	0.396	0.590	0.072	10.090	10.190	9.940
Result 95%	0.401	0.640	0.078	10.100	10.220	9.600
Result GA	0.426	0.578	0.074	10.130	10.190	9.960

Upper part: Period of application and estimated interval of parameter values (Upper/lower boundary). Lower part: Parameter calibration by ABC—results as percentile for each parameter—and parameter calibration by GA.

The resulting parameter values are determined by applying the ABC algorithm as described above. The resulting posterior distribution of parameter values $p\left( {\Theta |D} \right)$ is computed as frequency distribution from the accepted samples and stated in Table 1—lower part. The parameter values are additionally estimated by means of GA using the same lower–upper boundaries for the parameter search space. While slight differences are to be expected in the results due to the difference in the formulation of the error function, the results of the GA match the 50-percentile value of the ABC scheme with a mean relative deviation of 2.2%. We conclude that the simple ABC scheme is a suitable choice for parameter estimation.

### True infection dynamics

**Figure 4:**
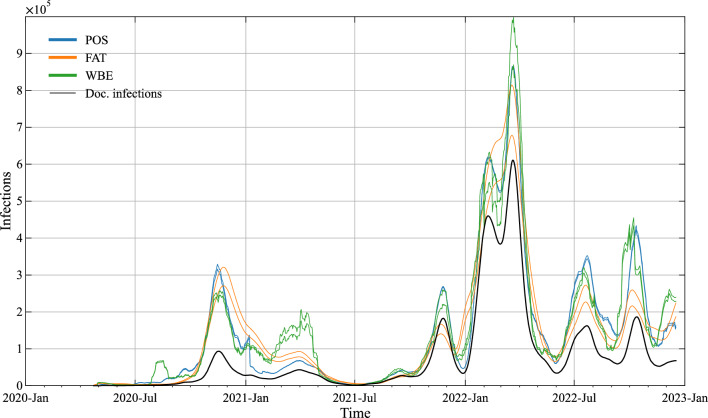
Estimated interval of true infections by means of the 3 models POS, FAT and WBE. Uncertainty in the estimates is plotted by using the 5 and 95 percentile values from ABC. The timeline of documented infections is plotted for comparison.

Figure 4 plots the resulting timelines of true infections of the three models by applying—for each—both the 5 and 95 percentile values of $p\left( {\Theta |D} \right)$. It is visually obvious that the uncertainty in the model estimates is small for all 3 models. The maximum relative deviation computed from the percentile values as: $max\frac{{\left| {5\% - 95\% } \right|}}{50\% }$ are 0.07, 0.18 and 0.16 for POS, FAT and WBE. As the uncertainty is negligible for practical purposes, we apply only the 50 percentile values of $p\left( {\Theta |D} \right)$ for further analysis.

Figure 4 further makes obvious that all 3 models give fairly coinciding results for estimating true infections—which is further corroborated by statistical metrics of similarity (Table 2). For the POS model the already mentioned change in the counting procedure of tests around 1st January 2021 introduces disturbances in the estimate of true infections. It is also to be noted that the FAT model is failing during the last period of occurrence of the Delta variant (Nov. 2022) as the model predicts the total infection numbers to be lower than the documented ones (*I* < *I*_*d*_). This shortcoming of the FAT model could be easily solved by further refinement of the parameter *IF*_*R*_ over the timeline. However, as the available survey data for parameter estimation is limited, we refrain here from doing so.

**Table 2:** Pairwise similarity of the model estimates of true infections.

	POS-FAT	POS-WBE	FAT-WBE
Euclidian	1,607,074	1,426,732	1,759,529
RMSE	51,626	45,833	56,524
MAPE	0.42	0.46	0.38
MSIM	0.81	0.83	0.80
R^2^	0.91	0.93	0.89

Table 2 plots different metrics to explore the pairwise similarity of the resulting timelines of the three models. As metric we apply Euclidian distance $\left( {\sqrt {\sum\nolimits_{i = 1}^{n} {\left( {x_{i} - y_{i} } \right)^{2} } } } \right)$, root mean square error (RMSE), mean average percentage error (MAPE), metric mean similarity $\left( {MSIM = \frac{1}{n}\sum\nolimits_{i = 1}^{n} {1 - \frac{{\left| {y_{i} - x_{i} } \right|}}{{\left| {y_{i} } \right| + \left| {x_{i} } \right|}}} } \right)$ and coefficient of determination ($R^{2}$)—see e.g., Rauch et al.^25^. The results of all five metrics indicate a high pairwise similarity of all three models, with POS-WBE forming a cluster ($R^{2}$ = 0.93).

In the absence of further information on prevalence data the accuracy of the estimation can be increased by combining the 3 models. Exemplarily, Supplementary Table S2 online gives the parameter values according to the ABC method for an averaged model and Supplementary Fig. S3 online plots the resulting true infections.

### Timeline of effective reproduction number

**Figure 5:**
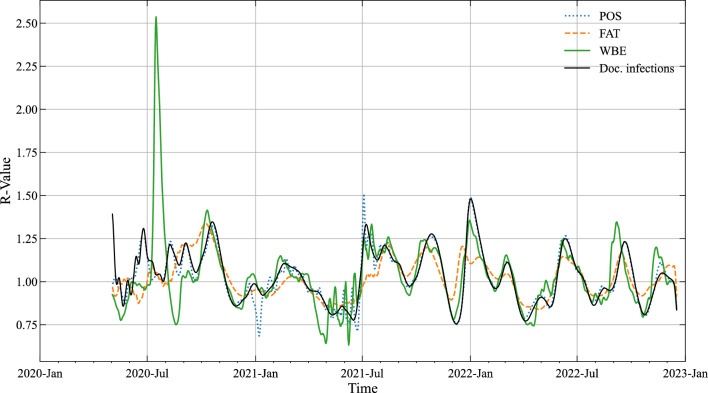
*R*-value of the estimated true infections (50 percentile values) with the 3 models and *R*-value computed for the documented infections.

As further test of reliability we compare the timelines of the effective reproduction number (*R*) derived from the results of the three models—Fig. 5. *R* stands for the average number of secondary infections generated by each new infection^55,56^ and is a standard parameter of pandemic management to track the infection progress. For computing *R* we first deconvolute the three timelines of true infections in order to derive the daily number of new infections for each model (*C*_*INF*_). For the actual calculation of *R* we use the simple method proposed by van der Heiden and Hamouda^57^ (denoted also as Robert Koch Institute method) by applying a serial interval value of 4 days:

9 $$ R_{t} = \frac{{\mathop \sum \nolimits_{{\hat{t} = t - 3}}^{t} C_{{INF, \hat{t}}} }}{{\mathop \sum \nolimits_{{\hat{t} = t - 7}}^{t - 4} C_{{INF, \hat{t}}} }} $$

*C*_*INF*_ stands for the (true) new infections at a given day $\hat{t}$*.* According to^57^ we smooth the resulting timeline of *R* by applying a moving average over 3 days. While there are more refined algorithms for *R* estimation available, Bsat et al.^56^ demonstrate that the Robert Koch method yields consistent results comparable with other methods.

In Fig. 5 the estimates of the 3 models of the reproduction number are plotted against the *R*-value computed for the documented new infections (*N*_*INF*_). The visual comparison the model estimates is quite convincing—with only one deviation of the WBE model at the early stage of the pandemic (Fig. 5). The test results for pairwise similarity are found in Supplementary Table S4 online. Uncertainty in the model estimates has been investigated as above by using the 5 and 95 percentile values for of $p\left( {\Theta |D} \right)$ but was found to be even smaller as for the true number of infections with the maximum relative deviation being 0.03, 0.01 and 0.06 for POS, FAT and WBE.

### Validation: case study Vienna

Typically, for model validation, either a portion of the timeseries is used for validation instead of training or the model is applied to a different dataset. Both approaches are problematic in this case: the FAT and POS data series contain time dependent effects on model parameters that make a split in training and validation data meaningless. Moreover, such a split would further reduce the already sparse information on true prevalence data, needed for parameter estimation. Regarding the use of a different dataset, it should be noted that the underlying secondary data (number of tests, fatalities, virus load, etc.) is heavily influenced by national pandemic management strategies such as number of diagnostic facilities or laboratory procedures. Therefore, data originating from outside Austria is likely to exhibit different statistical properties, making it unsuitable for validation purposes.

**Figure 6:**
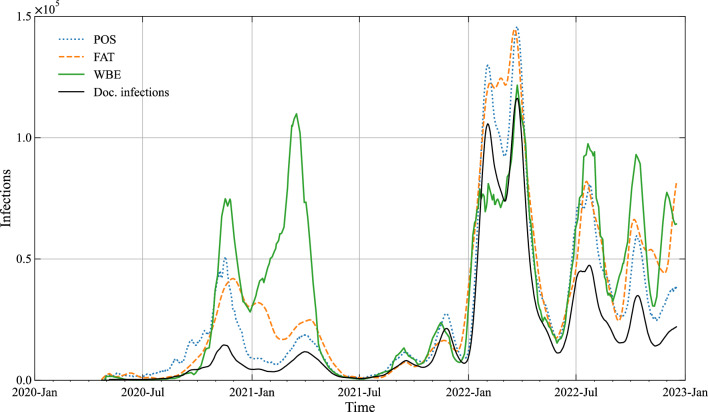
Estimated true infections by means of the 3 models POS, FAT and WBE for the city of Vienna. Parameters chosen as above, i.e. 50 percentile values from ABC for national data. The timeline of documented infections is plotted for comparison.

According to above, for validation we estimate the true infection dynamics for the case study Vienna (population 1.9 Mill.) by using the parameter values derived for the national data. Figure 6 reveals a fairly consistent estimate of true infection dynamics also for the case study, thus proving the general applicability of the approach. Still, there are two obvious differences in the WBE model results as compared to the documented infection cases: First, the WBE model computes a significant infection peak for the alpha variant (Spring 2021) which is not seen in the timeline of documented cases and second, the predicted infections are lower than the documented cases for the first omicron wave (BA1) in Spring 2022. Both deviations indicate differences in the monitored virus load in Vienna as compared to the averaged national signal. The deviation could be due to differences in monitoring and laboratory methods but also caused by external influences in the wastewater collection system.

### Forecast

**Figure 7:**
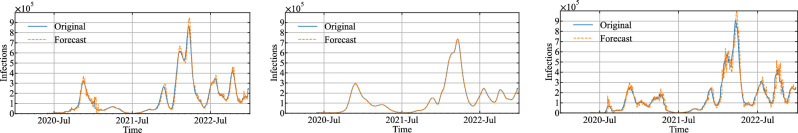
Rolling window analysis of the autoregressive model. The consecutive 7-day forecasts are plotted against the original data. Left: POS model, Middle: FAT model and Right: WBE model.

It is a crucial aspect of health management to anticipate future pandemic development for adequate strategies^58^. In order to test on the (short term) prediction capabilities of the 3 models developed herein, we apply the methodology that has been developed by Rauch et al.^25^ for the timeline of national true infections estimated with the 50 percentile values of the ABC approach. Despite complex data driven models are available for this task^59^, for testing the forecast capability it is sufficient to resort to a simple autoregressive (AR) model^60^. For testing, we choose a rolling window approach (see Rauch et al.^25^ for details) where we compare for each step in the whole timeline the 7-day prediction of the model with the actual value—here denoted original (Fig. 7). The prediction performance of the models is assessed with the metric root mean square error (RSME)—determined by summing up the error over the whole timeline. In order to eliminate trend and seasonality in the data we apply differencing prior to the modeling and back-transform the data after forecasting. The optimal order of the AR model is estimated by minimizing RSME.

As evident from Fig. 7 all 3 models are sufficient capable of short term (here 7 days) predictions. RSME values for the rolling window test values are determined as 27,884 (POS), 2442 (FAT) and 42,970 (WBE). The disturbance in the test counting around 1st Jan. 2021 (see also Fig. 1) causes likewise disturbances in the POS model predictions (Fig. 7 Left). The superior performance of the FAT model with respect to forecasting is likely due to the smoothing effect that is inherent in the model.

## Discussion

As there is no continuously measured ground truth data, it is impossible to identify model quality with respect to the estimation of true infections. Consequently, we cannot determine the optimal model for estimation of prevalence. Based on similarity testing, all three models investigated herein reveal fairly similar results and the validation case study proved the general applicability of the method. Advantage and disadvantages of the three models are seen as follows:

The POS model proved to be simple, yet robust against virus variants and model estimates could be derived with only one parameter value for the whole timeline. On the other hand, the POS model revealed a dependency on the number of tests and is sensitive to the estimated value of parameter *n*. In the investigated case of Austria (and also for the case study Vienna), the number of tests was exceptionally high as compared to most other countries, which potentially introduces a considerable positive bias for this model, in particular when it comes to cross-national comparisons. On the same note, the POS model is less suitable as surveillance tool as it is unlikely to maintain rigorous testing facilities in situations of low prevalence. E.g., in the Austrian situation the diagnostic testing of individuals stopped in July 2023.

The advantage of the FAT model is that it relies on a key metric of pandemic management, i.e. fatalities, without need of further monitoring. On the other hand, the fatality rate as key parameter of the model is not constant but varies with the occurrence of virus variants and vaccination. This feature was quite obvious for the occurrence of the Omicron variant, which resulted in the necessity to recalibrate the parameter. Further, the FAT model works only in a situation, when there are fatalities actually happening. Consequently, this model is likely to be too insensitive for early warning. Moreover, model results are dependent on a coherent and correct accounting of SARS-CoV-2 related fatalities, which is not an easy task in the early stage of a pandemic situation. An improvement could be to take into account hospitalization numbers instead of fatalities. And last, the signal is significantly delayed as compared to the actual situation due to the time lag between infection and death (app. 14 days).

The benefit of the WBE model is the high sensitivity of the signal and its reliability—as derived directly from the sought information, i.e., the true number of infected persons. This makes the model a suitable choice for surveillance. The shortcoming of the model is the time dependency of the summarizing parameter “corrected shedding load” *L*_*corr*_ that is determined by virus variants. Following qualitative information from the literature^42^ three parameters had to be introduced for the phases Ancestral, Alpha/Delta and Omicron. One point to consider is the uncertainty in the signal that is introduced by differences in test procedures and laboratory methods in the monitoring. The sensitivity of the model to the signal became obvious in the case study Vienna. Last, the WBE model could be improved with deeper knowledge on fecal shedding and use of sewer network parameters such as length, residence time and sewage temperature.

For estimating prevalence, all three models have a shared advantage: the underlying data inherently includes information on non-pharmaceutical interventions and vaccination. Effects therefrom on undercounting are considered in the parameter test positivity rate (P_+_) in the POS model and in the parameter infection fatality rate (*IF*_*R*_) in the FAT model. Since the WBE model utilizes the virus load from infected persons as its source, it inherently incorporates the impacts of non-pharmaceutical interventions and vaccination.

As mentioned already in the introduction there are several potential alternatives available for estimating prevalence. Capture-recapture methods are likewise based on secondary data (more specifically: documented infections and death counts) but—in the common parameter less formulation—lack in flexibility to adapt the resulting model to changing conditions in the course of the pandemic. A different approach is given by Richard’s curve and generalized logistic models, which have been widely used in epidemiological modeling to describe the spread of infectious diseases over time^61–63^. The methods apply sigmoidal asymmetrical growth models and provide a versatile framework for modeling non-linear relationships between predictors and response variables. As being based on incidence data, Richard’s curve and generalized logistic models result in cumulative incidence estimates but not directly in prevalence prediction. Also, the simulation of the entity of a pandemic including several waves, requires recalibration of the model or the use of several curves, each capable to describe individual waves^63^. Therefore, while these alternatives offer potential advantages, careful consideration of the specific context and requirements of the prevalence estimation task is necessary before their adoption.

## Conclusion

In the present study, we systematically investigated the suitability of three parameterized models to estimate the true number of infections (also denoted as prevalence estimation) from secondary data. As (secondary) input data the models use either the number of positive tests per day (POS model), the number of fatalities (FAT model) or the virus signal monitored from the wastewater stream (WBE model). The analysis was made for the case of Austria in the period April 2020 to December 2022, thus covering the bulk of the pandemic occurrence in Austria. To provide a coherent information along the timeline it was necessary to condense the signal towards national data. For validation the method has been applied to the case study Vienna—using the parameters found for the national situation. Key findings are as follows:

- As there is no ground truth data available for the true number of infections, the quality of model predictions cannot be rigorously assessed with metrics. However, similarity testing revealed fairly similar results for all three models investigated herein and the validation case study proved the general applicability of the method.
- Approximate Bayesian Computation is a simple but efficient tool for estimation of the distribution of parameter values. The 50 percentile of the post distribution values are matching the results from standard parameter estimation with genetic algorithms, thus corroborating the applicability of the ABC scheme.
- All three investigated models proved to be suitable to estimate the true number of infections. None of them is seen as superior, but advantages/shortcomings depend on the case study at hand. This indicates that all three datasets contain similar information.
- Uncertainty in the model estimates as computed by the 5 and 95 percentile values from the ABC approach was found to be quite small for the resulting number of true infections and insignificant for the estimated R-value.
- All three models allow for adequate short-term forecasting over 7 days. Best forecasting performance is exhibited by the FAT model due to inherent data smoothing.
- Despite its simplicity, the POS model gives convincing results in our case study, but requires a high number of tests for robustness.
- The FAT model works well in a pandemic situation but requires a coherent and correct accounting of SARS-CoV-2 related fatalities. Also, the signal is delayed for app. 14 days as compared to the actual situation.
- The WBE model gives a reliable signal as derived directly from the (true number of) infected persons, thus making it a suitable choice for pandemic surveillance. As a shortcoming the model is sensitive to case and variant specific differences in viral load.

While this study has a focus on SARS-CoV-2 we also wish to emphasize its relevance for other viral diseases, e.g., Noro- or Influenzavirus^64^. Early warnings and epidemiological predictions based on sound models also for these viruses and others may help in local, regional or national prevention.

### Supplementary Information

Supplementary Information.

## Data Availability

The datasets used and analysed during the current study are available from the corresponding author on reasonable request.
