# Supplementary material for: Estimating actual SARS-CoV-2 infections from secondary data
Source: Sci Rep. 2024 Mar 20;14:6732. doi: 10.1038/s41598-024-57238-0 (PMC10954653; doi:10.1038/s41598-024-57238-0)
Supplement: Supplementary file 1 — Supplementary Information. [file 41598_2024_57238_MOESM1_ESM.docx]

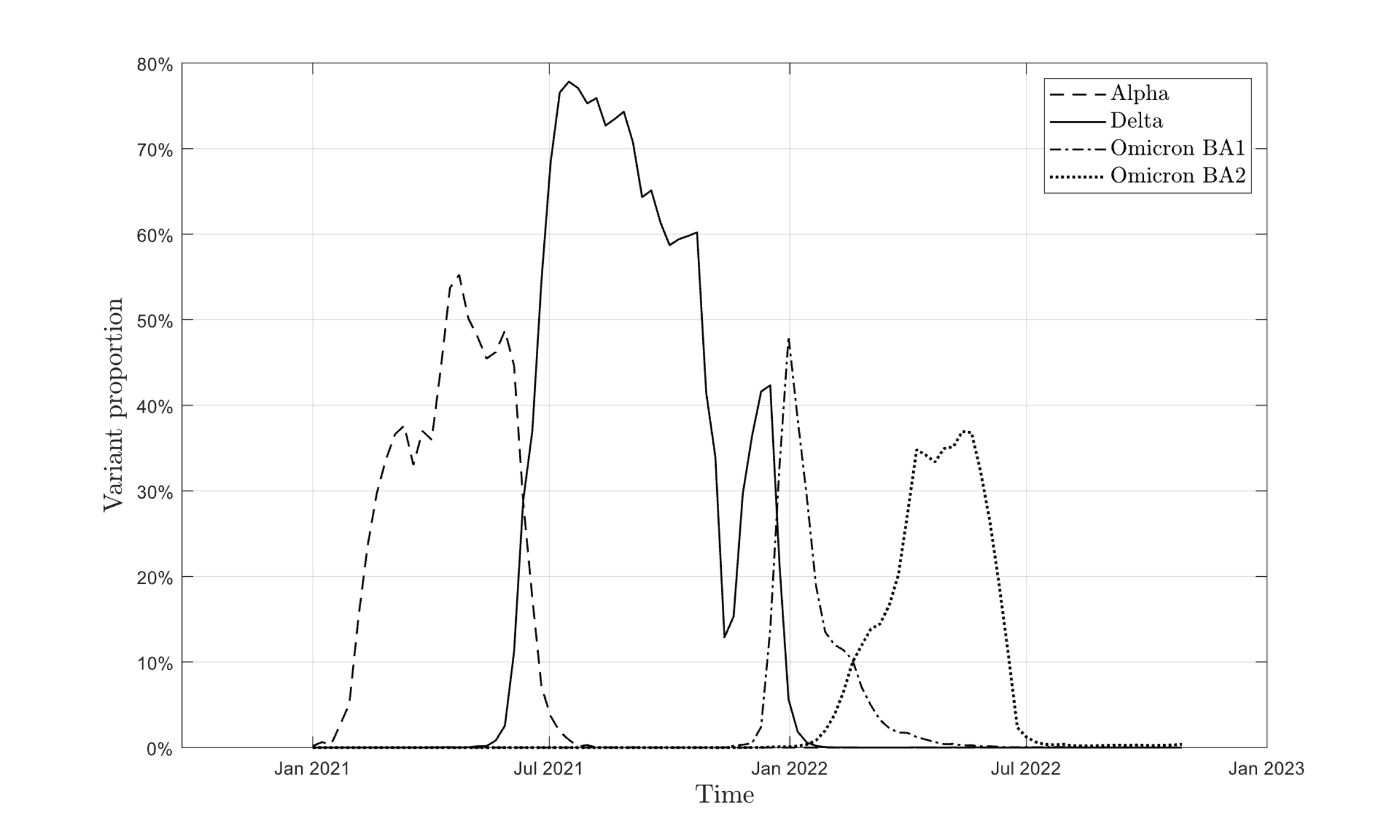


Supplementary Figure S1: Occurrence of dominant variants expressed as fractions of documented active Infection cases.

Supplementary Table S2: Averaged model: Parameters of the three models POS, FAT and WBE with parameter L_corr_ in log_10_ units. Upper part: Period of application and estimated interval of parameter values (Upper/lower boundary). Lower part: Parameter calibration by ABC – results as percentile for each parameter.


Supplementary Figure S3: Estimated true infections by means of the 3 individual models POS, FAT and WBE and the averaged model (Combined). Parameters are estimated as 50 percentile values from ABC.

Supplementary Table S4: Left: Pairwise similarity of the model estimates of R-values and Right: pairwise similarity with R-value of documented new infections (N_INF_)
